# Is routine serum TSH testing useful in the evaluation of primary subjective tinnitus? A case–control study

**DOI:** 10.3389/fneur.2026.1973811

**Published:** 2026-10-26

**Authors:** Aphrodite Mavrogenis, Stefani Maihoub, Panayiota Mavrogeni, András Molnár

**Affiliations:** 1 Bajcsy–Zsilinszky Hospital, Budapest, Hungary; 2 Maihoub ENT Clinic, Limassol, Cyprus; 3 Faculty of Health Sciences Doctoral School, University of Pécs, Pécs, Hungary; 4 Institute of Languages for Specific Purposes, Semmelweis University, Budapest, Hungary

**Keywords:** hearing levels, subjective tinnitus, thyroid dysfunction, tinnitus severity, TSH levels

## Abstract

**Objective:**

To investigate whether serum thyroid-stimulating hormone (TSH) levels are associated with primary subjective tinnitus and whether TSH provides clinically relevant information for the assessment of tinnitus.

**Materials and methods:**

This case–control study included 557 patients with primary subjective tinnitus and 431 tinnitus-free controls without known thyroid disorders. All participants underwent otorhinolaryngological examination and serum TSH measurement. Patients with tinnitus additionally underwent comprehensive audiological assessment, including pure-tone audiometry, psychoacoustic tinnitus matching, and assessment of tinnitus-related handicap using the Tinnitus Handicap Inventory (THI). Differences in TSH levels between the groups were analysed using the Mann–Whitney *U* test. Linear regression and Bayesian analyses were performed to evaluate associations between TSH and tinnitus-related parameters. Receiver operating characteristic (ROC) curve analysis was used to assess the ability of TSH to discriminate between participants with and without tinnitus.

**Results:**

Median TSH levels were 1.80 mIU/l (IQR 1.23–2.56) in the tinnitus group and 1.73 mIU/l (IQR 1.21–2.50) in the control group, with no significant difference between the groups (*p* = 0.63). Bayesian analysis provided moderate evidence in favour of the null hypothesis regarding the difference in TSH levels between the two groups (BF₀₁ = 4.459). TSH was not significantly associated with total THI score, hearing level, tinnitus intensity, age, or symptom onset in linear regression analyses. Bayesian regression analyses likewise provided evidence against a meaningful contribution of TSH to tinnitus-related parameters. ROC analysis demonstrated no clinically useful ability of TSH to discriminate between participants with and without tinnitus (AUC = 0.509, 95% CI 0.471–0.547, *p* = 0.640).

**Conclusion:**

These findings suggest that routine TSH measurement alone is unlikely to provide clinically relevant information for the assessment or management of primary subjective tinnitus in patients without known thyroid disease. However, these findings should not be extrapolated to patients with confirmed thyroid dysfunction or interpreted as a recommendation against thyroid function testing when clinical signs, symptoms, or other findings suggest underlying thyroid disease. Further studies incorporating comprehensive thyroid function assessment, including FT4, FT3, and thyroid autoantibodies, are warranted to clarify whether specific aspects of thyroid dysfunction may be relevant to selected tinnitus populations.

## 1 Introduction

Tinnitus is the perception of sound when no external source is present, affecting around 14% of adults. Persistent and bothersome tinnitus can significantly disrupt sleep, concentration, mood, and overall quality of life (1). Furthermore, there is a notable correlation between tinnitus and psychiatric symptoms, particularly depression and anxiety (2). There are two main types of tinnitus: primary tinnitus, which has no identifiable underlying cause and may occur with or without sensorineural hearing loss, and secondary tinnitus, which is linked to a specific medical condition (3). The assessment of tinnitus should extend beyond otological findings and include relevant non-otological and systemic factors (4). Systemic disorders, such as thyroid dysfunction, can be important considerations when evaluating potential secondary tinnitus. Thyroid dysfunction is a leading endocrine disorder, primarily due to thyroid autoimmunity, such as Hashimoto’s thyroiditis or Graves’ disease, in iodine-replete populations (5). In Europe, the prevalence of thyroid dysfunctions is approximately 3.82%, while prevalence of undiagnosed thyroid dysfunctions can be around 6.71%, with Hypothyroidism being the most common in both (6). A commonly used initial test for assessing thyroid function is the serum thyroid-stimulating hormone (TSH) level (7). Longitudinal studies conducted in populations have shown that the incidence of tinnitus is higher following diagnoses of both hypothyroidism and hyperthyroidism (8, 9). A cross-sectional study has identified associations between annoying tinnitus and low TSH levels (10). However, another study found no independent link between TSH and tinnitus but concluded that free thyroxine (FT4) levels were independently associated with a higher prevalence of tinnitus (11). The differing results observed in thyroid diseases, thyroid hormone levels, and study populations suggest that a clear and consistent relationship between serum TSH levels and tinnitus has not yet been established. This uncertainty limits the available evidence for routinely testing TSH levels in patients with primary subjective tinnitus and highlights the necessity for further targeted research in this area.

Given the uncertainty regarding the relationship between serum TSH levels and tinnitus, this study aimed to evaluate whether TSH levels differ between patients with primary subjective tinnitus without known thyroid disease and tinnitus-free controls, and whether TSH levels are associated with tinnitus-related clinical parameters. In particular, we sought to determine whether routine TSH measurement provides clinically useful information for the assessment of primary subjective tinnitus in the absence of a known thyroid disorder. The study was not designed to evaluate tinnitus associated with confirmed thyroid dysfunction.

## 2 Materials and methods

### 2.1 Study cohort

This study included 557 patients with primary subjective tinnitus and 431 participants in a control group. The basic characteristics of the study population are summarised in Table 1. Patients in the primary subjective tinnitus group were over 18 years old and experiencing persistent subjective tinnitus, regardless of any association with sensorineural hearing loss, and had provided their consent to participate in the study. The exclusion criteria eliminated individuals with secondary tinnitus (such as those diagnosed with Ménière’s disease, otosclerosis, or vestibular schwannoma), as well as patients with a history of hypo- or hyperthyroidism, other autoimmune or hormonal disorders, or those currently taking thyroid hormone medication. Patients with pulsatile tinnitus or hyperacusis were excluded from the study. Control participants were recruited from individuals undergoing otorhinolaryngological examinations for reasons unrelated to tinnitus or hearing complaints, including routine screening examinations. Participants were eligible if laboratory results including TSH measurements were available. Individuals reporting hearing complaints or tinnitus were excluded from the control group. A history of thyroid disorders was assessed through a detailed medical history and a thorough review of the available medical records. Participants with a documented or reported history of thyroid disease were excluded. Both groups underwent a thorough otorhinolaryngological examination and laboratory testing. The tinnitus group received specialised management from an experienced doctor in tinnitus care. Additionally, this group underwent a comprehensive examination, which included brain MRI and carotid-vertebral ultrasonography when necessary. They also received a detailed audiological assessment, as outlined below.

**Table 1:** Basic parameters of the study population.

Parameter	Tinnitus group (*n* = 557)	Control group (*n* = 431)	*p*-value
Age (median years; IQR, Q1–Q3)	51 (22; 42–64)	50 (15; 40–60)	0.06*
Sex (men/women)	292/265	204/227	0.11**
TSH levels (median mIU/l; IQR, Q1–Q3)	1.8 (1.3; 1.23–2.56)	1.73 (1.29; 1.21–2.50)	0.63*
Tinnitus location			
Right, *n* (%)	127 (22.4%)		
Left, *n* (%)	171 (30.7%)		
Bilateral, *n* (%)	259 (46.9%)		
Tinnitus intensity (median dB; IQR, Q1–Q3)	30 (20; 20–40)		
HL (median dB; IQR, Q1–Q3)	35 (32.5; 15–47.5)		
Tinnitus onset (median months; IQR, Q1–Q3)	12 (3–42)		
Total THI scores	40; (34.5; 24–58.5)		
THI category, *n* (%)			
Slight	86 (15.4%)		
Mild	176 (31.6%)		
Moderate	147 (26.4%)		
Severe	93 (16.7%)		
Catastrophic	55 (10%)		

dB, decibel; HL, hearing level; Hz, Hertz; IQR, interquartile range; mIU/l, milli-international units per litre; Q1, first quartile; Q3, third quartile; THI, Tinnitus Handicap Inventory; TSH, Thyroid-Stimulating Hormone. Statistical analyses were performed using the Mann–Whitney *U* test (indicated by *) and the Chi-squared test (indicated by **). The significance level was set at *p* < 0.05. Complete data were available for the variables included in each analysis. Tinnitus-specific variables were assessed only in the tinnitus group. Formal audiological assessments were not performed in controls; therefore, their hearing status was unavailable.

Written informed consent was obtained from all participants who agreed to participate in the study. The investigation was conducted in accordance with the Declaration of Helsinki and approved by the Hungarian ETT TUKEB (approval number: BM/29805–3/2025, approval date: March 11, 2026).

### 2.2 Pure-tone audiometry and tinnitus matching

Prior to the audiological assessments, all participants received a detailed otorhinolaryngological examination comprising otoscopy, tympanometry, and acoustic reflex testing. These procedures were used to identify and rule out potential middle-ear or other conductive abnormalities. Audiological measurements were then conducted by a trained audiological assistant and included pure-tone audiometry and tinnitus psychoacoustic matching. Hearing thresholds were obtained in a sound-attenuated booth with a GSI Pello Clinical Audiometer (Grason Stadler, Inc., Milford, CT, USA). Air-conduction thresholds were measured from 125 to 8,000 Hz, whereas bone-conduction thresholds were obtained from 250 to 4,000 Hz. Masking was applied during bone-conduction measurements when indicated by the audiological findings. Sensorineural hearing loss was classified according to the criteria established in the 1995 recommendations of the Committee on Hearing and Equilibrium of the American Academy of Otolaryngology–Head and Neck Surgery (12).

For participants reporting tinnitus, psychoacoustic pitch and loudness matching was completed on the same day as the audiometric examination. Pitch matching was performed across the 125–8,000 Hz frequency range, with the procedure initially presenting a 1,000-Hz tone. Participants compared the externally presented tone with their tinnitus and indicated whether the stimulus was perceived as higher or lower in frequency. The test frequency was subsequently adjusted iteratively until the participant considered it to correspond most closely to the perceived tinnitus pitch. Once the frequency match had been established, tinnitus loudness was determined at that frequency using 1-dB increments. The resulting pitch and loudness matches were recorded manually on the corresponding audiograms.

### 2.3 TSH level assessments

Serum TSH levels were measured by clinical laboratories as part of routine clinical care, using the same methods described below. Blood samples were collected into native serum tubes, and TSH concentrations were determined using the ADVIA Centaur® TSH3-Ultra II (TSH3ULII) assay on an ADVIA Centaur® XP/XPT automated immunoassay system (Siemens Healthineers). The assay is a third-generation chemiluminescent immunoassay designed for the quantitative measurement of TSH in human serum. The assay employs a sandwich immunoassay format using monoclonal anti-TSH antibodies. The detection system incorporates fluorescein isothiocyanate (FITC)-labelled anti-TSH antibodies, paramagnetic particles coupled to anti-fluorescein antibodies, and an acridinium-ester-labelled anti-TSH signal reagent. The chemiluminescent signal generated during the reaction is measured by the analyser, with the resulting relative light units (RLU) directly related to the TSH concentration in the sample.

For each determination, 100 μL of sample was required. The assay has a reported analytical measurement range of 0.008–150.000 μIU/mL (mIU/l). The manufacturer-reported reference interval for euthyroid adults is 0.550–4.780 μIU/mL (mIU/l). All results were validated by the laboratory performing the TSH measurement, and the treating doctor carefully reviewed them.

### 2.4 Self-reported tinnitus severity (Tinnitus Handicap Inventory)

To evaluate the impact of tinnitus on daily functioning, participants completed the Tinnitus Handicap Inventory (THI) in Hungarian. The THI consists of questions organised into three categories: functional, emotional, and catastrophic. Participants can respond to each question with ‘yes’ (4 points), ‘sometimes’ (2 points), or ‘no’ (0 points). The total THI score is calculated by summing the points from the subscales, which indicates the severity of tinnitus’s impact on daily life. Based on the THI results, tinnitus handicap can be classified into five levels: no handicap (0–16 points), mild (18–36 points), moderate (38–56 points), severe (58–76 points), and catastrophic (78–100 points) (13).

### 2.5 Statistical analysis

Data processing was conducted using IBM SPSS Statistics version 25.0 (IBM Corporation, Armonk, NY, USA). The normality of the data was assessed using the Shapiro–Wilk test, which indicated a non-normal distribution. Therefore, the Mann–Whitney *U* test was employed to identify statistically significant differences, and continuous variables were reported as medians along with their interquartile ranges (IQRs). The chi-squared test was used to analyse categorical data. Linear regression models were fitted to evaluate the associations between TSH levels and tinnitus-related parameters. Spearman’s rank correlation test was used to assess potential correlations. To assess the robustness of the associations, multivariable linear regression models were fitted with TSH as the predictor of interest, adjusting for age, sex, and hearing level (HL), where applicable. For the model in which HL was the dependent variable, adjustment was made for age and sex only. Analyses were based on participants with complete data for the variables included in each model. Bayesian analyses were performed alongside conventional frequentist analyses to quantify the relative evidence supporting the null and alternative hypotheses. Bayesian analyses were conducted using IBM SPSS Statistics version 25.0, with the default settings retained and no prior settings manually modified. For the Bayesian independent-samples analysis, the default settings included the assumption of unequal group variances, diffuse prior distributions for the variance and group means, and Rouder’s method for Bayes factor estimation. Posterior estimates and 95% credible intervals (CrIs) were used to characterise the posterior distribution. For Bayesian linear regression, Bayes factors were estimated using the default Zellner–Siow (JZS) method, with the null model as the reference model. The default reference prior was used for posterior characterisation. The full regression models included TSH and the covariates appropriate to each outcome and were compared with an intercept-only null model. Bayes factors were interpreted according to their magnitude and direction, with BF₁₀ values below 1 indicating evidence in favour of the null hypothesis relative to the alternative hypothesis. Receiver operating characteristic (ROC) curve analysis was performed to assess the discriminatory performance of TSH levels in distinguishing patients with tinnitus from controls. The area under the curve (AUC), its 95% confidence interval (CI), and the corresponding *p*-value were determined. The optimal TSH cut-off was selected using the Youden index, and the corresponding sensitivity and specificity were calculated. In addition, TSH concentrations were categorised as below, within, or above the laboratory reference interval (0.550–4.780 mIU/l), and the numbers and proportions of participants in each category were calculated separately for the tinnitus and control groups. A *p*-value of less than 0.05 was considered statistically significant for frequentist analyses.

## 3 Results

Table 1 presents the basic parameters of the study population.

Table 1 shows that there were no significant differences in age and sex between the tinnitus and control groups, ensuring that the two groups are statistically comparable. Tinnitus was primarily observed to be left-sided or bilateral. The median hearing levels (HL) indicate that most participants experienced mild to moderately severe sensorineural hearing loss. Additionally, the median duration since the onset of symptoms suggests that most cases are chronic. Finally, the median total THI scores indicate that participants most frequently experience mild or moderate handicaps.

In the next phase of the investigation, the TSH values between the tinnitus and control groups were compared (Figure 1).

**Figure 1:**
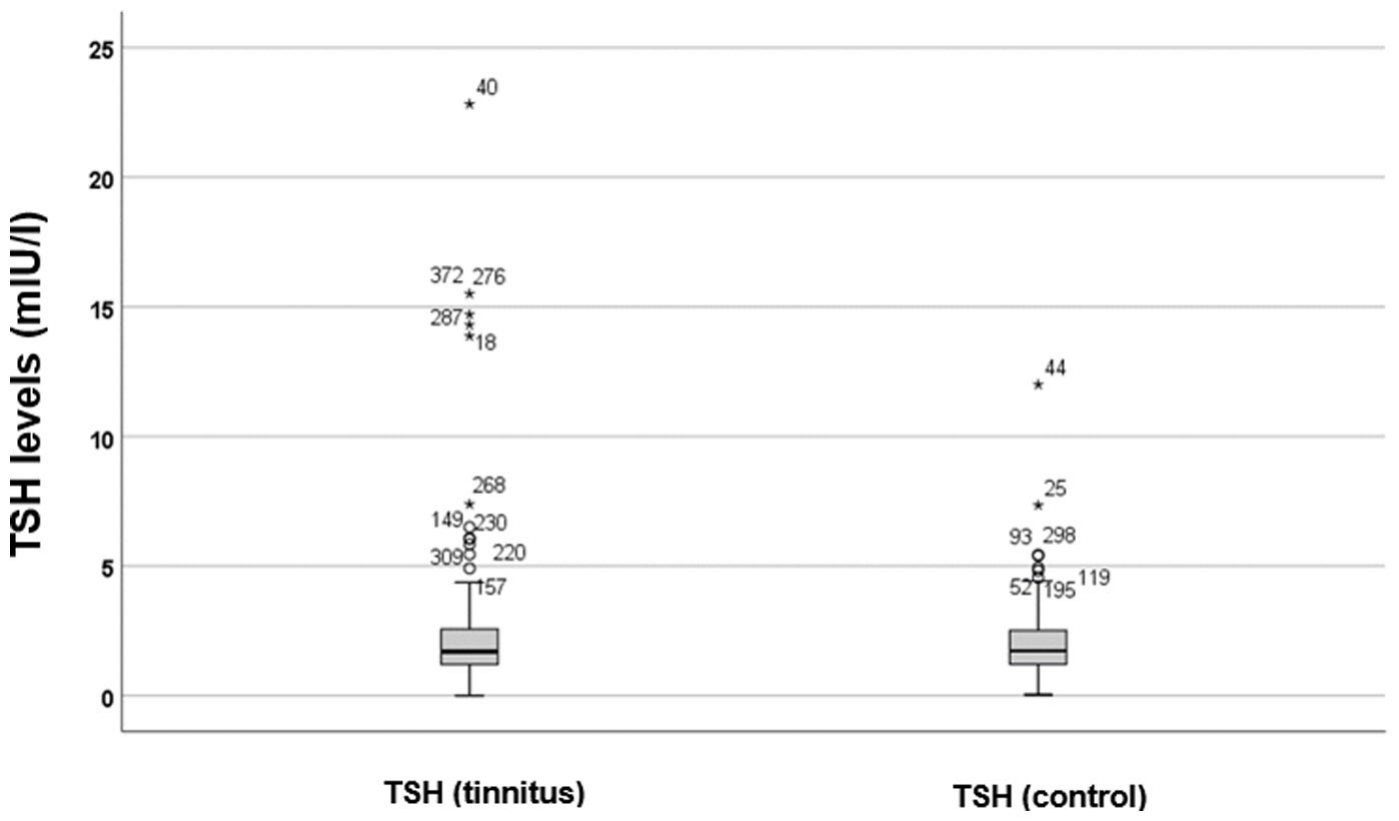
Boxplot illustrating TSH levels in the tinnitus and control groups. The boxes represent the middle 50% of the data, while the whiskers indicate the upper and lower 25%. The black line that divides each box represents the median values. The circles, asterisks and numbers depict the outliers. TSH, Thyroid-Stimulating Hormone; mIU/l, milli-international units per litre. Statistical differences were analysed using the Mann–Whitney *U* test (*p* < 0.05).

As shown in Figure 1, there is no clear difference in TSH levels between the tinnitus group and the control group. This finding is supported by the statistical analysis, which revealed no significant difference in TSH levels between the two groups (*p* = 0.63; *z*-score = 0.53).

To analyse the true absence of differences in TSH levels, a Bayesian Independent Samples Test was conducted (Table 2).

**Table 2:** Bayesian independent sample test (TSH levels).

Bayes factor independent sample test						
	Mean difference	Pooled std. error difference	Bayes factor	t	df	*p*-value
Tinnitus vs control group	−0.170	0.098	4.459	−1.729	986	0.084

Posterior distribution characterisation for independent sample					
	Mode	Posterior mean	Variance	95% CrI	
				Lower bound	Upper bound
Tinnitus vs control group	−0.170	−0.170	0.008	−0.350	0.011

CrI, credible interval; df, degrees of freedom; std., standard. The significance level was set at *p* < 0.05. BF₀₁ denotes the Bayes factor in favour of the null hypothesis relative to the alternative hypothesis. The reported BF₀₁ = 4.459 corresponds to BF₁₀ ≈ 0.224.

The Bayesian independent-samples analysis estimated a mean difference in TSH levels of −0.170 (95% CrI: −0.350 to 0.011). As the 95% CrI included zero, the analysis provided no clear evidence of a difference in TSH levels between the tinnitus and control groups. Furthermore, the Bayes factor (BF₀₁ = 4.459) indicated that the observed data were approximately 4.5 times more likely under the null hypothesis than under the alternative hypothesis, providing evidence in favour of the null hypothesis. This provides moderate evidence in favour of the conclusion that there is no meaningful difference in TSH levels between tinnitus patients and the controls.

To evaluate the associations between TSH levels and tinnitus-related parameters, both conventional linear regression and Bayesian regression analyses were performed (Tables 3, 4).

**Table 3:** Multivariable linear regression analyses of the association between TSH and tinnitus-related outcomes.

Dependent variable	Adjustment variables	*B*	SE	Standardised *β*	*p*-value
Total THI score	Age, sex, HL	0.039	0.926	0.003	0.967
HL (dB)	Age, sex	−0.218	0.563	−0.017	0.699
Tinnitus intensity (dB)	Age, sex, HL	−0.056	0.504	−0.005	0.911
Symptom duration (months)	Age, sex, HL	−0.501	1.464	−0.016	0.733

*B*, unstandardised regression coefficient; dB, decibel; HL, hearing level; SE, standard error; THI, Tinnitus Handicap Inventory; TSH, thyroid-stimulating hormone. Models evaluate the association between TSH and each outcome after adjustment for the listed covariates. The significance level was set at *p* < 0.05.

**Table 4:** Bayesian linear regression model.

Bayes factor model (linear regression)					
	Bayes Factor	*R*	*R-*squared value	Adjusted *R*-squared value	Std. error of estimate
Total THI scores	0.000184	0.106	0.011	−0.005	23.839
HL (dB)	5.30 × 10^16^	0.453	0.206	0.200	17.676
Tinnitus intensity (dB)	9.04 × 10^29^	0.698	0.487	0.479	13.414
Symptom onset (months)	25125.213	0.313	0.098	0.089	45.998

dB, decibel; HL, hearing level; std., standard; THI, Tinnitus Handicap Inventory.

Bayesian linear regression analyses were performed to evaluate the association between serum TSH levels and tinnitus-related parameters. The model including tinnitus intensity showed the highest explanatory power (*R*^2^ = 0.487; adjusted *R*^2^ = 0.479), followed by the model including HL (*R*^2^ = 0.206; adjusted *R*^2^ = 0.200). The model including symptom onset showed a lower explanatory power (*R*^2^ = 0.098; adjusted *R*^2^ = 0.089), whereas the model based on the total THI score yielded a very low *R*^2^ value (0.011). The corresponding Bayes factors and model fit statistics are presented in Table 4.

To assess the robustness of the findings, multivariable linear regression analyses were performed to evaluate the associations between TSH levels and tinnitus-related outcomes, adjusting for age, sex, and HL where applicable (Table 3). TSH levels were not significantly associated with total THI scores (*B* = 0.039, SE = 0.926; *p* = 0.967), HL (*B* = −0.218, SE = 0.563; *p* = 0.699), tinnitus intensity (*B* = −0.056, SE = 0.504; *p* = 0.911), or symptom duration (*B* = −0.501, SE = 1.464; *p* = 0.733). These findings indicate that the absence of significant associations between TSH and the examined tinnitus-related outcomes persisted after adjustment for relevant covariates.

In the final step of the investigation, a ROC curve analysis was conducted to evaluate how TSH levels predict tinnitus (Figure 2; Table 5).

**Figure 2:**
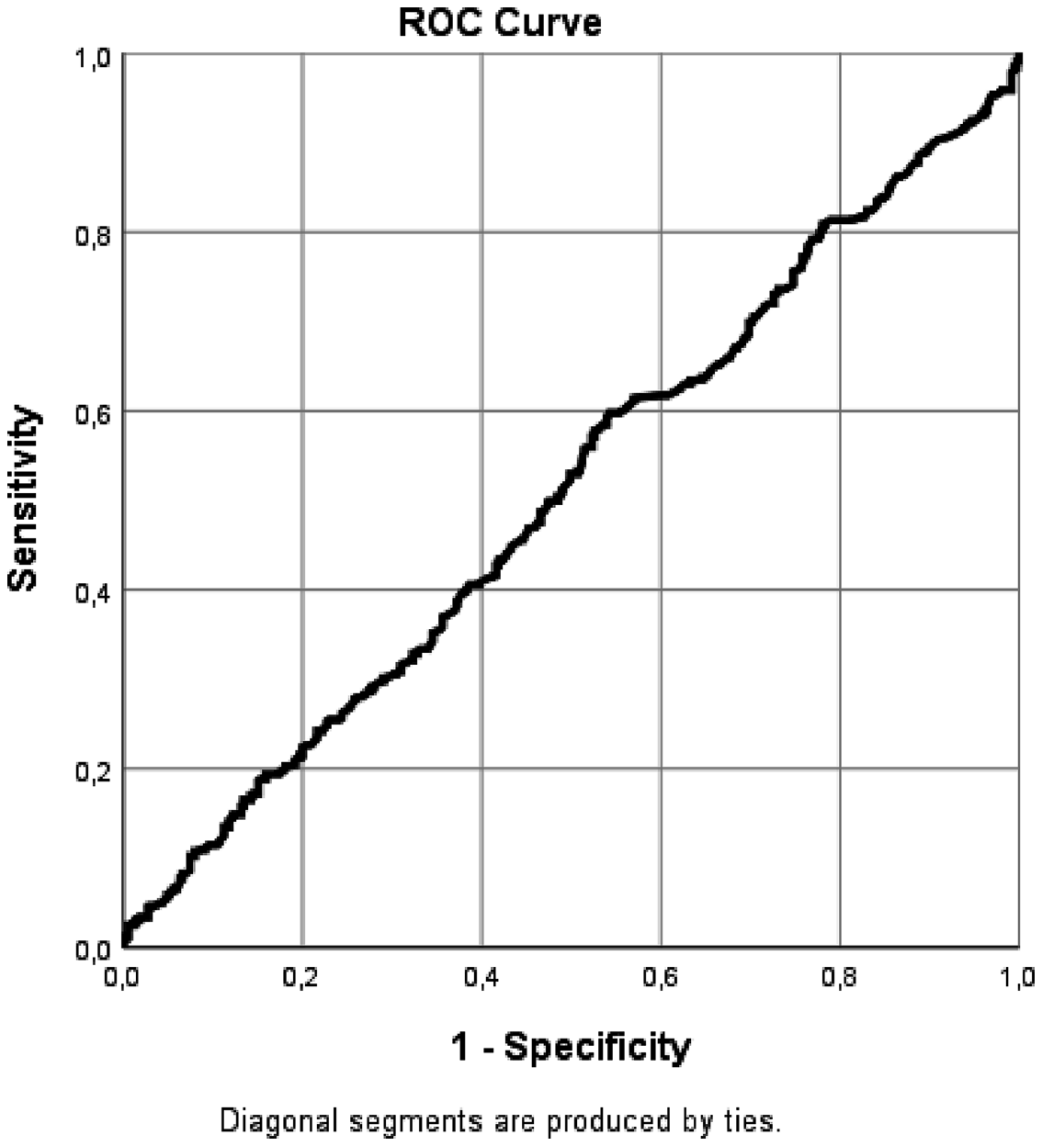
ROC curve analysis for predicting tinnitus based on TSH values. ROC, receiver operating characteristics.

**Table 5:** ROC analysis of TSH levels for distinguishing patients with tinnitus from controls, and distribution of TSH concentrations relative to the laboratory reference interval (0.550–4.780 mIU/l).

ROC analysis	
Parameter	Result
AUC (95% CI)	0.509 (0.471–0.547)
*p*-value	0.640
Optimal cut-off (mIU/l)	1.59
Sensitivity (%)	59.8
Specificity (%)	45.8
Youden index	0.055

TSH relative to the reference interval (0.550–4.780 mIU/l)		
TSH category	Tinnitus group (*n* = 557)	Control group (*n* = 431)
Below reference interval, *n* (%)	41 (7.4%)	16 (3.7%)
Within reference interval, *n* (%)	499 (89.6%)	408 (94.7%)
Above reference interval, *n* (%)	17 (3.1%)	7 (1.6%)
Total outside reference interval, *n* (%)	58 (10.4%)	23 (5.3%)

The optimal cut-off was determined using the Youden index. AUC, area under the curve; CI, confidence interval; mIU/l, milli-international units per litre; ROC, receiver operating characteristics; TSH, Thyroid-Stimulating Hormone. The significance level was established at *p* < 0.05.

ROC curve analysis demonstrated poor discriminatory performance of TSH levels in distinguishing patients with tinnitus from controls (AUC = 0.509, 95% CI: 0.471–0.547; *p* = 0.640). The optimal cut-off determined using the Youden index was 1.59 mIU/l, corresponding to a sensitivity of 59.8% and a specificity of 45.8% (Youden index = 0.055). These findings indicate that TSH levels do not provide clinically useful discrimination between tinnitus cases and controls.

Using the laboratory reference interval of 0.550–4.780 mIU/l, TSH concentrations were below the reference interval in 41 (7.4%) patients with tinnitus and 16 (3.7%) controls, and above the reference interval in 17 (3.1%) patients with tinnitus and 7 (1.6%) controls. TSH concentrations were within the reference interval in 499 (89.6%) patients with tinnitus and 408 (94.7%) controls. Overall, TSH concentrations outside the reference interval were observed in 58 (10.4%) patients with tinnitus and 23 (5.3%) controls (Table 5).

To investigate potential differences in TSH levels according to tinnitus-related burden, TSH levels were compared between patient groups defined by total THI scores. The results are presented in Figure 3.

**Figure 3:**
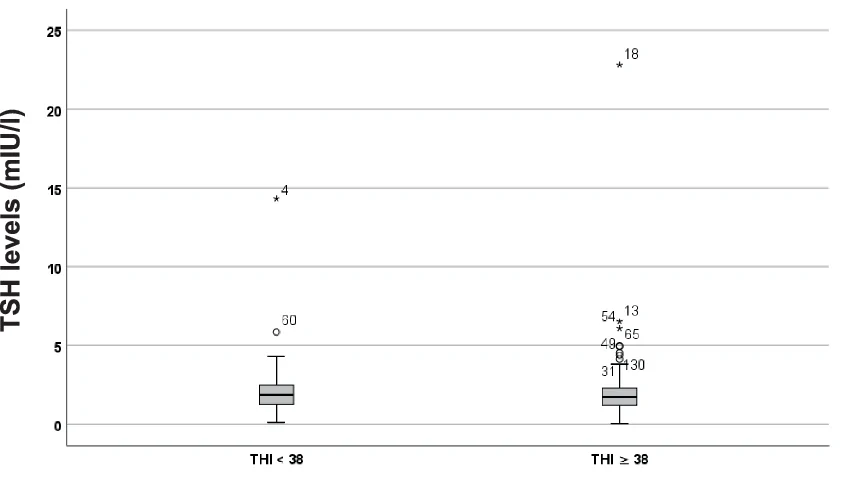
Boxplot illustrating TSH levels according to total THI scores. Higher tinnitus-related burden was defined as a THI score ≥ 38, corresponding to moderate, severe, or catastrophic handicap. The boxes represent the middle 50% of the data, while the whiskers indicate the upper and lower 25%. The black line that divides each box represents the median values. The circles, asterisks, and numbers depict the outliers. mIU/l, milli-international units per litre; THI, Tinnitus Handicap Inventory; TSH, Thyroid-Stimulating Hormone. Statistical differences were analysed using the Mann–Whitney *U* test (*p* < 0.05).

As shown in Figure 3, TSH levels did not differ significantly between the two groups defined according to tinnitus-related burden (*p* = 0.19; *z*-score = 1.31). Additionally, Spearman’s rank correlation analysis revealed no significant correlation between TSH levels and total THI scores (ρ = −0.060, *p* = 0.270).

## 4 Discussion

This study examined the potential association between TSH levels and primary subjective tinnitus in a large group of patients and a control group. The initial findings revealed no statistically significant difference in serum TSH levels between the tinnitus and control groups, and no significant associations between TSH levels and tinnitus-related parameters. Bayesian analyses provided complementary evidence regarding the group difference and the contribution of predictors in regression models. Importantly, multivariable linear regression analyses showed that TSH levels were not significantly associated with total THI scores, HL, tinnitus intensity, or symptom duration after adjustment for age, sex, and relevant clinical covariates. Similarly, TSH levels did not differ significantly between patients with lower and higher tinnitus-related burden, and no significant correlation was found between TSH levels and total THI scores. ROC analysis further demonstrated poor discriminatory performance of TSH for distinguishing patients with tinnitus from controls. Taken together, these findings suggest that TSH measurement alone provides limited clinically relevant information for the assessment of primary subjective tinnitus in individuals without known thyroid disease. Recent literature has highlighted the importance of distinguishing tinnitus from tinnitus disorder in terms of their clinical and neurobiological characteristics (14, 15). In light of this distinction, we additionally examined TSH levels according to tinnitus-related burden based on total THI scores; however, neither the comparison between groups nor the correlation analysis revealed a statistically significant association between TSH levels and tinnitus-related burden.

The management of tinnitus in everyday practice remains limited. Therefore, it is crucial to identify easily measurable parameters that can enhance diagnosis and management. TSH is a commonly assessed parameter for evaluating thyroid function; however, its relevance to tinnitus is not well established. Other biochemical parameters, including serum 25-hydroxyvitamin D3 levels, have also been investigated in relation to tinnitus occurrence and severity (16).

Currently existing literature contains sparse findings in terms of thyroid dysfunction in relation to tinnitus. Previous investigations have explored the relationship between both hypo- and hyperthyroidism and auditory disorders, including tinnitus. For instance, a scoping review concluded that there is a significant correlation between hypothyroidism and auditory dysfunction as well as tinnitus (17). It is important to note that this review did not specifically focus on primary subjective tinnitus, as it also included secondary cases of tinnitus. Another study found that tinnitus (26%) and hearing loss (20%) were common symptoms in patients with hypothyroidism, with these symptoms occurring more frequently in the hypothyroid group compared to controls. Among the types of hearing loss observed, sensorineural hearing loss was the most commonly associated with hypothyroidism. Furthermore, after undergoing thyroxine replacement therapy, most cochleovestibular symptoms showed improvement. A statistically significant enhancement in hearing levels, as measured by the pure-tone audiometry, was also noted following treatment (18). Hashimoto’s thyroiditis is a major cause of hypothyroidism in adults (19). In this condition, circulating thyroid autoantibodies, particularly anti-thyroid peroxidase (anti-TPO) and anti-thyroglobulin (anti-TG) antibodies, may contribute to immune-mediated dysfunction of the cochleovestibular system, potentially involving T-cell-mediated mechanisms (20). One population-based study found a 1.35 HR increased risk of tinnitus in the hypothyroidism cohort (8). However, another study did not find an association between tinnitus and hypothyroidism (21). The association between thyroid function and tinnitus also appears to be inconsistent across individual thyroid hormone parameters. A recent analysis of data from the National Health and Nutrition Examination Survey (NHANES) found that lower FT4 levels were significantly associated with a higher prevalence of tinnitus, whereas neither free triiodothyronine (FT3) nor TSH showed a significant association with tinnitus. These findings further suggest that the relationship between thyroid dysfunction and tinnitus may be complex and cannot be readily attributed to abnormalities in a single thyroid hormone or regulatory parameter (11). Consequently, the evidence regarding the association between hypothyroidism and tinnitus remains limited and inconsistent. In our selected cohort of patients with primary subjective tinnitus without known thyroid disease, serum TSH levels were not significantly associated with tinnitus-related outcomes. These findings address the potential value of TSH measurement in this specific population and do not exclude a relationship between tinnitus and confirmed thyroid dysfunction.

In terms of hyperthyroidism, a population-based longitudinal study demonstrated a significant association between hyperthyroidism and an increased risk of tinnitus in both men and women. Interestingly, the risk was most pronounced during the first 3 years following the diagnosis of hyperthyroidism and subsequently decreased (9). However, it should be noted that in that study, specific TSH and thyroid hormone levels were not available, as both hypothyroidism and tinnitus were identified based on ICD diagnostic codes. In contrast, our study measured serum TSH in all participants and examined its relationship with primary subjective tinnitus in individuals without known thyroid disease. However, because FT4, FT3, and thyroid autoantibodies were not assessed, our findings do not provide a comprehensive evaluation of thyroid function or the potential relationship between confirmed thyroid dysfunction and tinnitus. Another study found that subclinical hyperthyroidism was associated with an increased risk of bothersome tinnitus, an association that was observed primarily in women in the subgroup analysis (10). Although previous studies have suggested an association between hyperthyroidism, low TSH levels, and tinnitus, the available evidence remains inconsistent.

Thyroid hormones may influence auditory development and function through thyroid hormone receptor signalling in the cochlea, particularly through THRA and THRB, which contribute to cochlear maturation and inner hair cell function. Local regulation of thyroid hormone availability by deiodinases, as well as effects on auditory synaptic development, may also be involved (22–24). Thyroid dysfunction may additionally affect inner-ear perfusion through adrenergic mechanisms (17). Although these mechanisms provide biological plausibility for a relationship between thyroid dysfunction and auditory symptoms, they cannot be directly evaluated in the present study, which focused on serum TSH levels rather than comprehensive thyroid function or cochlear thyroid hormone signalling.

This study has several strengths. First, it involved a relatively large population, providing a substantial sample size for investigating the potential association between serum TSH levels and tinnitus. Second, the research applied strict inclusion and exclusion criteria, specifically excluding patients with secondary tinnitus and those who had previously been diagnosed with thyroid disorders or received thyroid hormone treatment. This focus allowed for the analysis of primary subjective tinnitus in individuals without known thyroid disease. TSH concentrations were measured using a standardised third-generation automated immunoassay conducted after an overnight fast, offering an objective biochemical measure. Lastly, the study employed both frequentist and Bayesian statistical approaches, combined with regression and ROC curve analyses. This methodology enabled the evaluation of non-significant findings not only in terms of statistical significance but also regarding the strength of evidence supporting the absence of a clinically meaningful association.

Several limitations should be considered when interpreting these findings. First, the observational case–control design precludes causal inference and does not establish whether alterations in thyroid function precede the development or progression of tinnitus. Second, TSH was measured at a single time point. As TSH levels may fluctuate over time and throughout the day, a single measurement may not fully reflect an individual’s usual or long-term thyroid status. Third, the investigation focused exclusively on serum TSH; FT4, FT3, and thyroid autoantibodies were not assessed. Consequently, comprehensive thyroid function could not be characterised, and potential associations between tinnitus and other aspects of thyroid dysfunction may have been missed. Fourth, participants with previously diagnosed thyroid disorders or receiving thyroid hormone treatment were excluded. Therefore, the findings may not be generalisable to patients with known or confirmed thyroid dysfunction, including overt or subclinical thyroid disease. Although thyroid history was assessed through medical history-taking and review of available records, previously undiagnosed thyroid conditions may have remained unidentified. Fifth, audiological assessments were not performed in the control group. Consequently, hearing status could not be directly compared between the groups, and undiagnosed hearing loss among controls cannot be excluded. This limits the assessment of baseline hearing comparability and may have influenced the interpretation of associations between TSH and hearing-related outcomes. In addition, recruiting controls from individuals undergoing otorhinolaryngological examinations for reasons unrelated to tinnitus or hearing complaints may have introduced selection bias and limited the generalisability of the findings. Finally, some tinnitus-related measures, including symptom severity and psychoacoustic characteristics, rely partly on participants’ subjective responses. Future prospective studies incorporating repeated TSH measurements, comprehensive thyroid function testing, and audiological assessment in both cases and controls are warranted to clarify the potential relationship between thyroid function and tinnitus.

## 5 Conclusion

In this large case–control study, serum TSH levels did not differ significantly between patients with primary subjective tinnitus and tinnitus-free controls. Furthermore, TSH levels were not significantly associated with tinnitus severity, hearing levels, tinnitus intensity, age, or symptom duration. Bayesian analyses provided additional evidence supporting the absence of a meaningful association between TSH and tinnitus-related parameters, while ROC analysis demonstrated no clinically useful discriminatory ability of TSH for identifying patients with tinnitus. These findings suggest that routine TSH measurement alone is unlikely to provide clinically relevant information for the assessment or management of primary subjective tinnitus in patients without known thyroid disease. However, these conclusions should not be extrapolated to patients with confirmed thyroid dysfunction and do not imply that thyroid evaluation is unnecessary in all patients with tinnitus. Comprehensive thyroid function testing remains appropriate when clinical signs, symptoms, or other findings raise suspicion of underlying thyroid disease. Further prospective studies incorporating comprehensive thyroid function testing, including FT4, FT3, and thyroid autoantibodies, are warranted to clarify whether specific aspects of thyroid dysfunction may be relevant to selected tinnitus populations.

## Data Availability

The raw data supporting the conclusions of this article will be made available by the authors, without undue reservation.
